# A new analgesic method, two-minute sciatic nerve press, for immediate pain relief: a randomized trial

**DOI:** 10.1186/1471-2253-8-1

**Published:** 2008-01-25

**Authors:** Jiman He, Bin Wu, Xianrong Jiang, Fenglin Zhang, Tao Zhao, Wenlon Zhang

**Affiliations:** 1 Biomedicine (TC), Chinese Academy of Sciences, Beijing, 100080 China & Rhode Island Hospital, Brown University, RI 02903, USA; 2 Renal Department, Anhui Province Hospital, Anhui Medical University, Hefei, 230001, China; 3 Department of Biology, Hefei Teachers College, Hefei, 230061, China; 4 Department of Oncology, Maanshan People Hospital, Maanshan, 243000, China; 5 Department of Emergency, Anhui Province Hospital, Hefei, 230001, China; 6 Department of Dentistry, Chuzou Zhongxiyi Hospital, Chuzou, 239000, China

## Abstract

**Background:**

Current analgesics have drawbacks such as delays in acquisition, lag-times for effect, and side effects. We recently presented a preliminary report of a new analgesic method involving a two-minute sciatic nerve press, which resulted in immediate short-term relief of pain associated with dental and renal diseases. The present study investigated whether this technique was effective for pain associated with other disease types, and whether the relief was effective for up to one hour.

**Methods:**

This randomized, placebo-controlled, parallel-group trial was conducted in four hospitals in Anhui Province, China. Patients with pain were sequentially recruited by participating physicians during clinic visits, and 135 patients aged 15 – 80 years were enrolled. Dental disease patients included those with acute pulpitis and periapical abscesses. Renal disease patients included those with kidney infections and/or stones. Tumor patients included those with nose, breast, stomach and liver cancers, while Emergency Room patients had various pathologies. Patients were randomly assigned to receive a "sciatic nerve press" in which pressure was applied simultaneously to the sciatic nerves at the back of both thighs, or a "placebo press" in which pressure was applied to a parallel region on the front of the thighs. Each fist applied a pressure of 11 – 20 kg for 2 minutes. Patients rated their level of pain before and after the procedure.

**Results:**

The "sciatic nerve press" produced immediate relief of pain in all patient groups. Emergency patients reported a 43.5% reduction in pain (p < 0.001). Significant pain relief for dental, renal and tumor patients lasted for 60 minutes (p < 0.001). The peak pain relief occurred at the 10 – 20^th ^minutes, and the relief decreased 47% by the 60^th ^minutes.

**Conclusion:**

Two minutes of pressure on both sciatic nerves produced immediate significant short-term conduction analgesia. This technique is a convenient, safe and powerful method for the short-term treatment of clinical pain associated with a diverse range of pathologies.

**Trial registration:**

Current Controlled Trials ACTRN012606000439549

## Background

Human beings have long suffered from pain caused by diseases. In medicine, pain is one of the most common reasons for patients to seek care. Analgesics used in current practice have drawbacks such as delays in acquisition and lag-times for effect after administration. In addition, many commonly used analgesics have considerable side-effects [1-4].

Many non-drug analgesic interventions have been used to help manage pain, including acupuncture, cryoanalgesia, transcutaneous electrical nerve stimulation (TENS), exercise, and music therapy, etc. [5-7]. Though literature reviews document the efficacy of some non-drug analgesic interventions [5,8,9], their use are usually restricted to some pain centers, and, the clinical effectiveness of some analgesic methods are controversial [10-12].

We recently published a preliminary report on a new analgesic method, a 2-min sciatic nerve press, which immediately relieved pain brought on by various dental and renal diseases [13]. The technique is simple, can be used any time, any place, immediately upon the onset of pain (including outside of a hospital setting). No side effects have yet been seen.

The present study examined whether the technique worked on more pathologies, and whether pain relief extended to up to one hour.

## Methods

### Setting and procedures

The study was a randomized, single-blinded, placebo-controlled, parallel-group trial consisting of 135 patients treated between October 2005 and May 2006 at four hospitals in China. The study was separately approved by each participating hospital – Anhui Province Hospital, Hefei, 230001, China (Approval date-June 2, 2005); Maanshan People Hospital, Maanshan, 243000, China (Approval date-May 20, 2005); Tongling Count Hospital, Tongling Count, 244100, China (approval date-October 18, 2005), and Chuzou Zhongxiyi Hospital, Chuzou, 239000, China (approval date-May 28, 2005). Written informed consent was obtained from each participating patient.

Patients were divided into "sciatic press" and "placebo press" groups. Instructions and explanations were equally provided to all patients. Patients were informed that the experiments were designed to test whether the methods were effective for pain relief. Patients were advised that they could discontinue participation in the study at any time without penalty, and that their healthcare treatment would not otherwise be affected.

After informed consent was obtained, the participating doctor(s) or assistants taught the patients how to evaluate pain using a visual analogue scale (VAS), where pain scored from "0" for no pain, to "10" for most pain. Thereafter, randomization of patients to the "sciatic press" or "placebo press" group was performed using the method of Random Permuted Blocks. The three stages of the test were then described to each patient: baseline pain rating, 2 min of leg pressure while lying down, and post-pressure pain rating at the specified times.

Two test designs were used. For Emergency patients, pain was rated once after pressure application. In contrast, renal, dental and tumor patients underwent the 60 min test in which pain was rated at 10-min intervals for a period of one hour after pressure application. In this context, the '0 min' point indicated the time at which the 2-min leg pressure stopped and the post-pressure pain rating stage commenced. The baseline pain rating and leg pressure stages were identical for the two types of tests.

**Figure 1:**
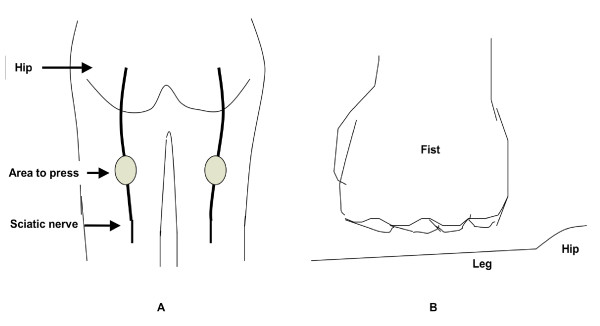
(A) The location of the pressure areas on the sciatic nerve. (B) The gesture for applying fist pressure.

The positions of sciatic nerve pressure and the fist gesture used for pressure application are shown in Figure 1. This manipulation technique has been previously described in detail [13]. Briefly, 2 min of pressure with the dorsal, proximal phalangeal surface of the fists (not the knuckles or finger tips) was applied simultaneously to the sciatic nerve sites or the placebo location on both legs. For the "sciatic nerve press", 11 to 20 kg of pressure was applied to the sciatic nerves on the back of the thighs with each fist, while patients lay prone. For the "placebo press", the same pressure was applied to a parallel spot on the front of the thighs, while patients lay supine. Patients then stood and rated their pain using a visual analogue scale table. The amount of force applied within the range of 11–20 kg depended on the patient body type, with heavily muscled large-bodied patients receiving higher pressures than thin patients. Doctors learned the pressure force applied by repeatedly pressing on a weighted balance.

### Participants

The inclusion criterion was any patient who was feeling pain during a clinic visit to the Emergency room, Renal, Dental, or Oncology Clinics. Exclusion criteria were age less than 15 years, emotional instability, or administration of another analgesic within 12 hours of the test. For enrolled patients, the dental diseases included acute pulpitis and periapical abscesses. The renal diseases included infections and/or stones, and the tumors included those of the nose, breast, stomach, and liver. While Emergency Room enrollees had various pathologies. No patients had previously been treated with the sciatic press method. For tumor patients, analgesics were discontinued 11–13 hours prior to the test, and the study began when patients reported they had moderate-to-severe pain.

The patient groups and characteristics are shown in Table 1. Of the 229 solicited patients, 57 refused to join and 37 were considered ineligible, leaving 135 patients aged from 15 – 80 years to participate in the study. Of those 135, two renal patients in the placebo group withdrew in the middle of the 60 min test stating that the pain was too severe to continue.

**Table 1:** Patients groups and characteristics at inclusion

	**Placebo press **(n = 70)	**Sciatic press **(n = 65)	**p value**
Emergency test			
Participants (n)	49	46	-
Male (%)	57.1%	67.3%	0.24
Age	41.9 ± 13.4	37.6 ± 12.7	0.12
Baseline VAS	7.1 ± 1.88	7.3 ± 1.63	0.49
			
60 min test in renal, dental and tumor patients			
Participants (n)	21	19	-
Renal	28.6%	21.1%	0.86
Dental	38.1%	42.1%	
Male (%)	42.9%	52.6%	0.54
Age	42.5 ± 18.3	41.6 ± 15.4	0.88
Baseline VAS	6.05 ± 1.36	6.39 ± 1.55	0.46

Age, gender and baseline VAS: mean (± SD), t-test.

### Statistical analysis

Renal, dental and oncology patients were analyzed as one group in terms of relief of pain. Emergency room patients were analyzed separately, and without pathology categorization. The baseline VAS scores and age of the participants were compared between the "sciatic press" and "placebo press" groups using t-tests. Categorical data were analyzed using chi-square, or Fisher Exact tests. In terms of pain relief, changes from baseline were assessed using paired *t*-tests for both groups. Comparisons with the "placebo press" groups were performed using an analysis of covariance procedure, with adjustment for baseline VAS score, gender and age. All tests were two-sided, and a p-value of < 0.05 was considered to indicate significance. All statistical analyses were performed using SPSS statistical software (release 13.0).

## Results

**Figure 2:**
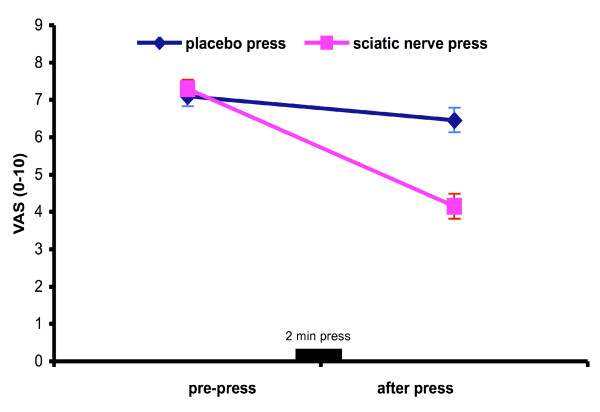
Pain relief in emergency patients. Data represent the mean VAS score (± SE) (p < 0·001).

The study included 95 Emergency Department patients with various pain-related conditions (Figure 2). Following application of the "sciatic press" technique, patients recorded a 43.5% decrease in the mean VAS pain scores (p < 0.001). The pain relief was significantly greater than with the "placebo press" (p < 0.001). Notably, over 70% of Emergency patients reported pain relief after the "sciatic press".

**Figure 3:**
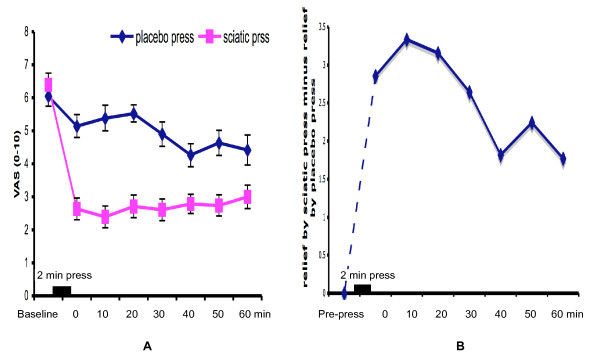
The 60 min test for the combined group of renal, dental and oncology patients. Data represent the mean VAS score (± SE). (p < 0·001 for all post-pressure points between the "sciatic press" and placebo groups).

To test the longer-term effectiveness of the "sciatic press" method, 40 patients with pain from tumors, dental and renal diseases were tested for a period of one hour (Figure 3). Pain data for the three patient groups were pooled, and demonstrated that mean VAS scores were lower at all time points (p < 0.001) for the "sciatic press" group compared to the placebo group (Figure 3A). Pain was reduced by 54.5% at the 10^th ^minute, and by 35.3% at the 60^th ^minute.

In the "placebo press" group, the VAS score was reduced by 11–15% within the first 10 min, and by 23.4–29.5% between the 40^th ^and 60^th ^minutes. Data were also expressed by subtracting "placebo press" values from "sciatic press" values and plotting the resultant value against time (Figure 3B). The analysis showed that the peak pain relief of this technique occurred at 10^th ^– 20^th ^minutes, with relief reduced 47% by the 60^th ^minute.

## Discussion

Mechanical pressure can cause nerve abnormalities. A number of studies have reported the effect of pressure on nerves using different force levels and time periods of up to several weeks [14-21]. In animal models, pressure studies usually involve surgically exposed nerves [16,17,22]. However, the present method uses a much shorter time period, two minutes for the pressure on the intact skin. Thus far, the method has been applied to over 600 subjects across 10 hospitals and universities, and there have been no reports of adverse side-effects. The type of acute pressure applied in this technique (11 – 20 kg by each fist (150 – 380 mmHg) for up to 2 min) is not uncommon in daily activities, especially for people involved in sports or heavy manual labor. However, nerve injuries as a result of this procedure remain a possibility, and must be taken into account in future studies. The present technique does not involve chronic pressure on nerves, of which even a very small amount could cause severe nerve dysfunction. For example, chronic pressure on the sciatic nerve by internal tension of the obturator muscle or through anatomical abnormalities in the piriformis muscle could cause pain [23-26]. Surgery to relieve the muscle pressure results in immediate pain relief [23,26-28].

The present study was single-blinded rather than double-blinded as the method is highly effective for pain relief, and doctors could easily identify the placebo or the active treatment during the test. After patients reported pain scores, patients themselves recorded their pain assessments on a VAS table, or in some cases, had the doctors record the VAS score. However, it would be a stronger study to have employed a blinded observer, which we will include in our future studies.

To our knowledge, no clinical studies have reported on the use of pressure stimulation of the anterior or posterior thigh area for pain relief. The study design ensured no patients had previously undergone this technique, and we believe the 'blinding' procedures remained secure during testing. The pressure of 11 – 20 kg with each fist was chosen based on pilot studies using Chinese patients. Such a pressure range may differ for Western populations given the difference in average body size.

The present study showed that Emergency patients had a mean baseline VAS score of 7.2, and that the "sciatic press" resulted in a 43.5% reduction in pain. Nearly 30% of these patients reported no pain relief. Similarly, our previous study showed that renal patients had a mean baseline VAS score of 7.7, that the sciatic press method resulted in a 52.2% pain reduction, and that 40% of renal patients felt no pain relief [13]. The previous study also showed that dental patients had a baseline VAS score of 6.4, and that the treatment resulted in a 66.4% reduction in pain [13], and these are similar results to those from tumor patients who had a baseline VAS score of 5.8 and pain reduction of 70.7% (tumor patient test data are not shown in the present report). These data suggest that the analgesic effect may correlate with the baseline pain score. However, the present study was not comprehensive enough to make such a statistical connection, and this hypothesis awaits further testing in future larger studies.

In the "placebo press" group undergoing the 60 min test, VAS scores decreased markedly in later time periods (23.4–29.5% between the 40th and 60th min) compared to the first 10 min (11–15% reduction). In this group, 25% of patients had pain at the early time points but not at later times. For this study setting, we cannot rule out the total or partial contribution of a placebo effect, or some anterior nerve stimulation to the late drop. Pain discontinuity could also be contributing partially.

Stimulation of peripheral nerves elevates the pain threshold [29-32]. According to the Gate Control Theory of Pain [33], stimulation of large-diameter afferent fibers can inhibit the transmission of nociceptive information from the dorsal horn to higher brain centers. This inhibition occurs rapidly and is thought to involve the wide dynamic range (WDR) neurons [34-36]. The resulting analgesic effect is considered to be a short-lasting, segmental inhibition of pain [37-39]. This theory may explain the rapid pain relief observed in some situations. However, the pain relief created by the present method is not limited to the segmental level, but occurs throughout the body. Also, significant pain relief lasted for 60 minutes. These observations suggest possible activation of multiple inhibitory systems. Another mechanism possibly involved is activation of the endogenous opioid system. Yao found that low frequency stimulation of the rat sciatic nerve increased the pain threshold by 50%, and that this effect was antagonized by Naloxone which suggested activation of the opioid system [40].

Pinch press of the rat sciatic nerve with a vascular clip (pinch force 120 g) caused attenuation of WDR neuron responses to various innocuous and noxious stimuli [41]. While the WDR neuron response to superficial peroneal nerve stimulation was shown to increase in cats when the sciatic nerve was under a clip pressure with a pinch force 180 g, the response was inhibited when using low frequency stimulation (0.2 Hz) [32]. In the present clinical study, despite it being likely that there were slight variations in the pressure location and pressure force applied, pain relief was consistently achieved in all test groups. The current findings are similar to those from our pilot studies in which mechanical pressure was applied at different locations along the sciatic nerves using methods other than fists (data not shown).

## Conclusion

Two minutes of pressure on both sciatic nerves can produce immediate and significant conduction analgesia. This procedure provides a convenient, safe and powerful method for the short-term treatment of clinical pain induced by diverse disease types.

## Competing interests

The author(s) declare that they have no competing interests.

## Authors' contributions

JH was the primary investigator, had full access to all data, and takes responsibility for the integrity of the data and the decision to submit the work for publication. BW participated in renal clinical tests and data analysis. XJ collaborated in the pilot study and the mechanism study. FZ collaborated in the technical aspects and in administrative support for tumor patient testing. TZ participated in Emergency room tests. WZ collaborated in clinical tests on dental patients and in data interpretation. All authors read and approved the manuscript.

## Pre-publication history

The pre-publication history for this paper can be accessed here:


